# Histoplasmosis of the External Auditory Canal

**DOI:** 10.7759/cureus.35644

**Published:** 2023-03-01

**Authors:** Michael Rockwell, Patrick Spiller, Areli K Cuevas-Ocampo, Alexandre Malek, Gauri Mankekar

**Affiliations:** 1 Medicine, Louisiana State University Health Sciences Center, Shreveport, USA; 2 Otolaryngology - Head and Neck Surgery, Louisiana State University Health Sciences Center, Shreveport, USA; 3 Pathology, Louisiana State University Health Sciences Center, Shreveport, USA; 4 Infectious Diseases, Louisiana State University Health Sciences Center, Shreveport, USA

**Keywords:** liposomal amphotericin b, amphotericin, external auditory ear canal, histoplasmosis, chatgpt

## Abstract

This report describes the diagnosis and treatment of a patient with a rare fungal infection of the external ear, as well as a review of the literature. A 76-year-old Caucasian gentleman from rural southern United States with diabetes and hypertension was referred to our clinic for intractable left otalgia, otorrhea, headaches, and an exophytic lesion in the left external ear since five months. There was no pertinent travel history. Biopsy by an outside otolaryngologist was inconclusive. Repeat biopsy under anesthesia revealed morphological characteristics consistent with histoplasmosis. Intravenous amphotericin B and later oral antifungal agent voriconazole led to improvement in symptoms. The clinical presentation resembled a malignancy. A high index of clinical suspicion, histologic confirmation with deep tissue biopsy, and culture are essential for diagnostic confirmation followed by treatment with systemic antifungals. A multidisciplinary team approach is necessary to manage this rare condition.

## Introduction

Histoplasmosis is a fungal infection caused by the dimorphic fungus *Histoplasma capsulatum*. It typically involves the lungs but can spread to other parts of the body such as the brain or the spinal cord or head and neck [1]. Isolated histoplasmosis of the external auditory canal (EAC) has not been reported previously. Histoplasmosis and pulmonary calcifications have been described as endemic to the Mississippi and Ohio River Valleys of North and Central America [2]. In 2020, the Centers for Disease Control and Prevention updated its map to include broader areas in the United States and populations at a higher risk due to immunosuppression [3]. Histoplasmosis is also known to occur in parts of Central and South America, Africa, Asia, and Australia. Host immune status and inoculum size are known to influence the severity and organs affected by histoplasmosis. Presentation can include life-threatening disseminated disease, pulmonary nodules, or ocular histoplasmosis syndrome, making it challenging to diagnose.

## Case presentation

A 76-year-old Caucasian male from rural Southern United States with a medical history of hypertension, hyperlipidemia, bilateral renal cysts, and chronic kidney disease was referred to our otolaryngology clinic for worsening left otalgia since five months. He rated the pain as greater than 10 on a 0-10 pain scale with no relief provided by acetaminophen or ibuprofen. He also reported left otorrhea, headache, and jaw pain for two weeks. He did not have a history of ear infections, surgeries, or travel. Head and neck examination revealed a 1.5-centimeter tender, ulcerative, and exophytic lesion involving the anterior-superior wall of the outer one-third of the left ear canal. The left tympanic membrane could not be visualized as the EAC lesion filled the ear canal and occluded its view. Weber examination lateralized to the left, and both right and left Rinne tests showed air conduction greater than bone conduction. There was no palpable cervical lymphadenopathy. Audiogram could not be performed due to the severity of the patient’s pain and discomfort. Biopsy by an outside otolaryngologist was inconclusive.

**Figure 1:**
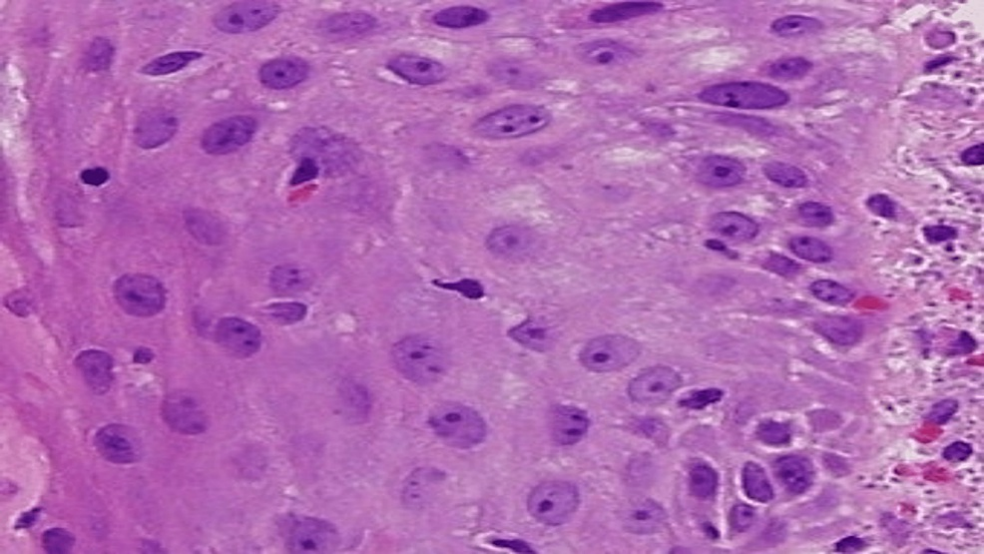
Squamous epithelium with underlying dermal diffuse infiltration by foamy histiocytes containing intracellular yeast (H&E stain x200)

**Figure 2:**
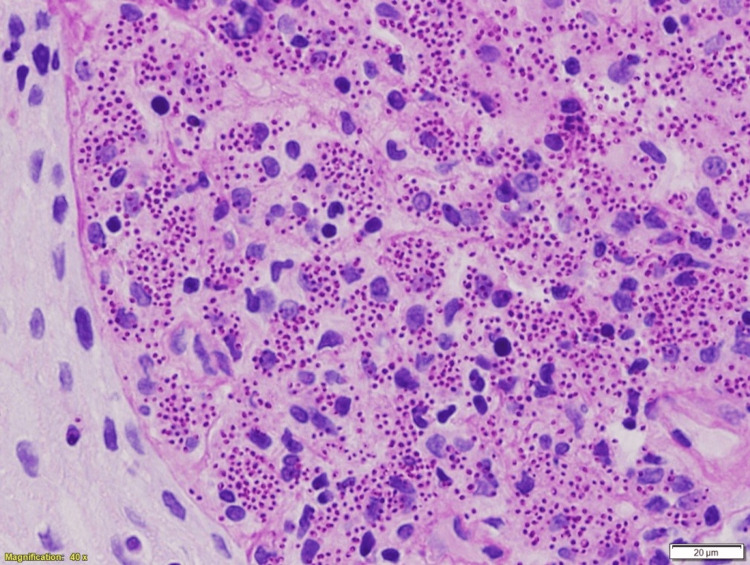
A higher magnification shows numerous PAS-positive intracellular yeast (PAS stain x400) PAS, periodic acid-Schiff stain

**Figure 3:**
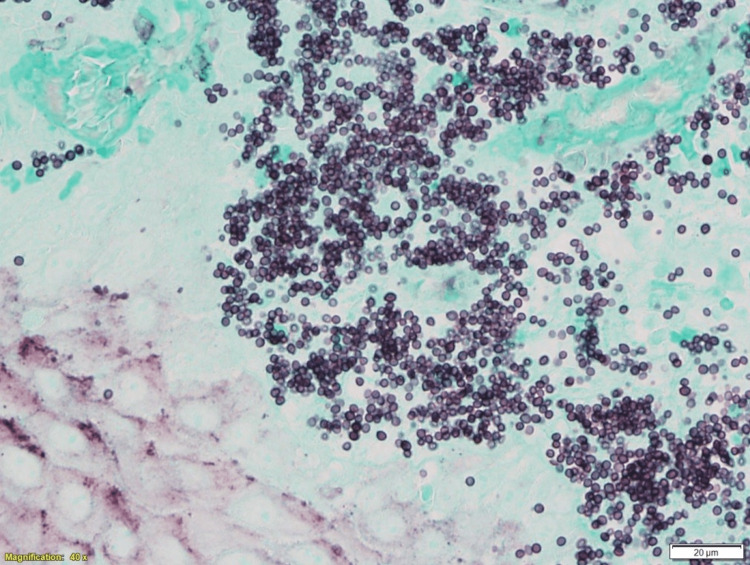
Intracytoplasmic GMS-positive fungal elements measuring 2-5 microns, compatible with Histoplasma sp. (GMS stain x400) GMS, Grocott-Gomori methenamine silver stain

On presentation to our clinic, the EAC lesion was biopsied with topical anesthesia. It showed subepithelial granulomatous inflammation with no evidence of malignancy. Microbiology showed few coagulase-negative staphylococcus. There was no growth on fungal culture. Computed tomography (CT) of the temporal bone revealed soft tissue density in the EAC without bony erosion or invasive features. As the biopsy was inconclusive and did not match the clinical presentation, he was taken to the operating room and placed under general anesthesia for an aggressive wide and deep excisional biopsy of the left ear canal lesion. Frozen section was inconclusive. Final pathology showed subepithelial granulomatous inflammation with intracellular yeasts measuring 2-5 microns that were positive for Grocott-Gomori methenamine silver stain (GMS) and periodic acid-Schiff stain (PAS) (Figures 1-3).

These morphological features were compatible with the diagnosis of histoplasmosis of the left EAC. The patient was referred to Infectious Disease (ID) clinic, where tests for serum *Histoplasma* antigen, blastomycosis antigen, and Cocci antibody were negative. Serum Histoplasmosis yeast was positive at 1:16 concentration indicating active Histoplasmosis infection. Human immunodeficiency virus (HIV), hepatitis C virus (HCV), and hepatitis B virus (HBV) screening was negative. X-ray of the chest performed at this time was normal. Five weeks after the CT scan, a magnetic resonance image (MRI) of the brain and head was performed, which showed interval worsening of the cutaneous lesion of the left EAC as compared to the prior CT performed. No signs of bone invasion or intracranial extension were noted.

Malignant neoplasm of the EAC was considered due to the severity of pain and clinical appearance of the lesion. However, biopsy of the EAC lesions revealed no evidence of malignancy, and CT scan did not show any invasive features. The diagnosis of bacterial skull base osteomyelitis was also considered; however, initial swabs and cultures of the left EAC showed no evidence of bacterial growth. Despite this, the presentation of otorrhea, inflammation, narrowing of the EAC, tenderness to palpation, and worsening pain at night which was out of proportion to the clinical findings led us to strongly consider skull base osteomyelitis caused by an unusual organism.

After histopathology confirmation, the patient was referred to the ID clinic and started on oral itraconazole. One week later, itraconazole was switched to voriconazole 200 mg twice daily due to a lack of symptomatic and clinical improvement. Two days later, the patient was admitted for increasing left otorrhea, otalgia, and headaches. Per ID recommendations, he was started on intravenous (IV) amphotericin. An ear wick for stenting the ear canal was placed. As the patient had a history of allergy to quinolones, sulfacetamide ear drops were prescribed. The patient had symptomatic improvement after a week of IV amphotericin. He was transitioned to oral voriconazole and discharged from the hospital. The patient was followed on an outpatient basis weekly for ear wick changes. After 5 weeks of oral voriconazole, the ear canal lesions resolved. The ear drops, ear wick, and oral antifungals were discontinued. The patient has been following up with us for the past eight months, is symptom-free, and has healed ear canal.

## Discussion

**Figure 4:**
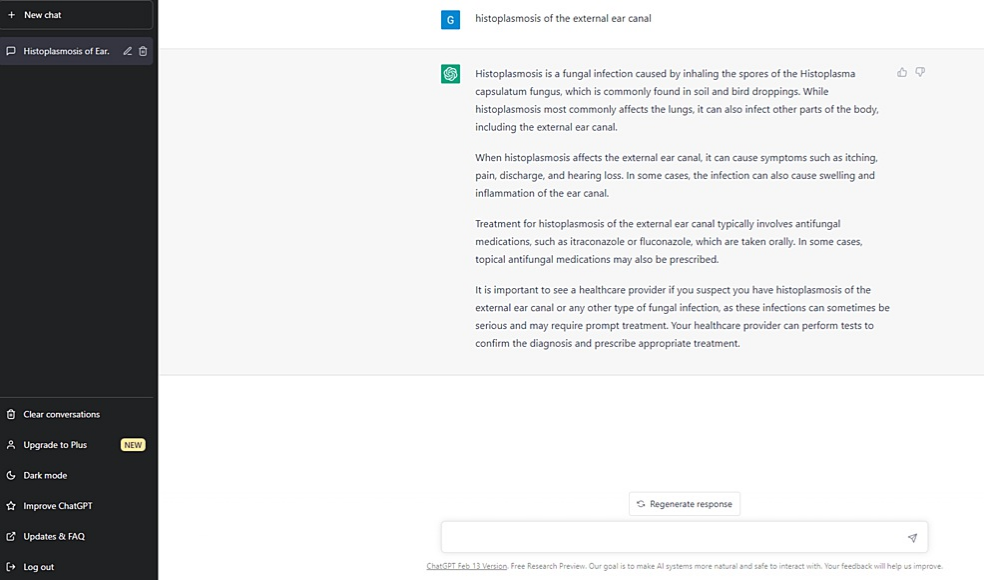
ChatGPT: histoplasmosis

**Figure 5:**
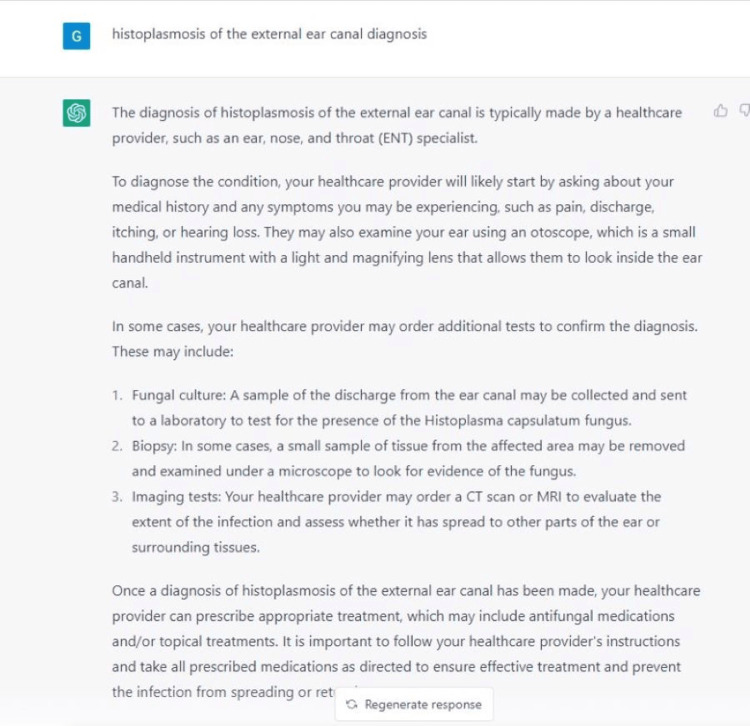
ChatGPT: histoplasmosis diagnosis

**Figure 6:**
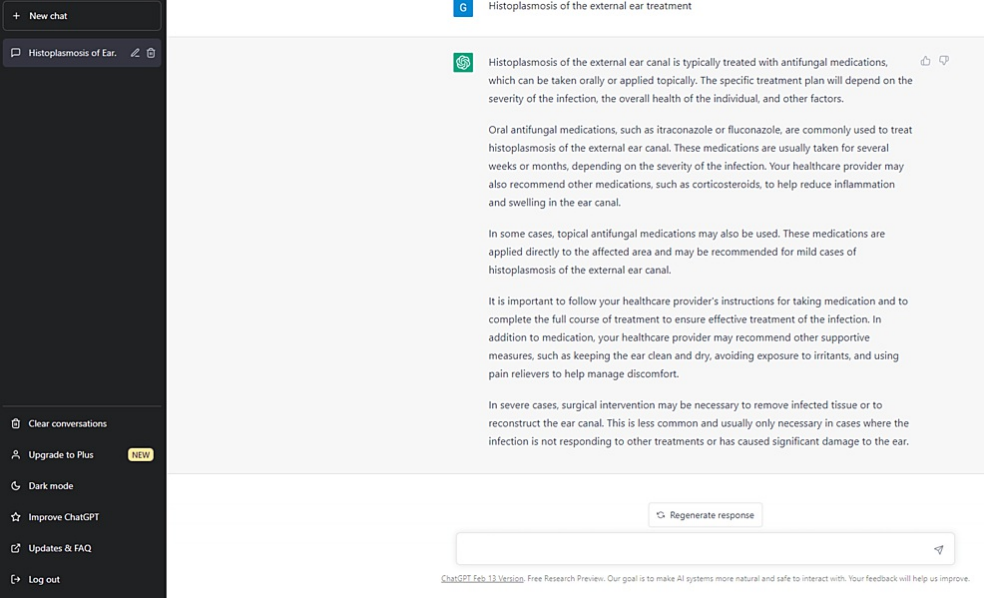
ChatGPT: histoplasmosis treatment

Histoplasmosis is a fungal infection caused by inhaling the spores of the *Histoplasma* capsulatum fungus [ChatGPT] (Figure 4). It is commonly found in the soil and bird droppings [ChatGPT] (Figure 4). Although histoplasmosis commonly affects the lungs, it can also infect other parts of the body including the external ear canal [ChatGPT] (Figure 4). It is known that histoplasmosis presentation can vary in severity depending upon the immune status of the host. Its wide range of manifestations creates a diagnostic challenge, as shown in this patient. Disseminated histoplasmosis can occur in immunocompetent patients, as the fungus spreads hematogenously through the reticuloendothelial system via infected macrophages. However, T cell-mediated immunity is normally able to contain the infection in immunocompetent hosts [4]. Symptomatic disseminated histoplasmosis typically occurs in immunocompromised hosts that are unable to mount an adequate cell-mediated immune response. Risk factors include HIV infection, transplant recipients, and autoimmune diseases [5]. With the exception of the pulmonary system, isolated single-organ infections are rarely reported [6]. Gurgel et al. [7] reported a case that presented initially with a 5-mm enhancing left internal auditory canal mass on MRI but later developed multiple neurologic deficits. Isolated oral ulcers with no disseminated infection are very uncommon but have been reported in a few cases [8,9]. Our patient had multiple medical conditions and had an isolated left EAC lesion without other organ involvement, which was confirmed by his normal X-ray of the chest and imaging studies of the brain. Diagnosis in challenging cases like these depends on a medical history, ear examination with an otoscope and microscope, fungal culture, biopsy of the ear canal lesion, and imaging studies [ChatGPT] (Figure 5). A high index of clinical suspicion along with histopathology can confirm the diagnosis. Imaging studies are necessary to exclude disseminated disease. MRI is superior to CT and more sensitive in determining the extent of the disease [10], and it was performed on our patient. Histoplasmosis has been associated with bird or bat droppings, but it should not be ruled out in patients without such exposures [5]. Our patient denied any exposure to bird or bat droppings. Histoplasmosis is typically treated with antifungal medications. The specific treatment plan depends on the severity of the infection and the overall health of the individual [ChatGPT] (Figure 6). Liposomal amphotericin B and oral itraconazole are standard drugs of choice for the treatment of histoplasmosis and help in achieving cure, as in our patient.

## Conclusions

Histoplasmosis presentation can be highly variable, and diagnosis can be challenging as described in our patient. Diagnosis and treatment require a high index of suspicion and a multidisciplinary team approach. Histoplasmosis has been considered to be endemic to certain regions and typically described as a disseminated disease. Isolated histoplasmosis of the ear canal, as seen in our patient, has not been reported.
